# Autologous and not allogeneic adipose-derived stem cells improve acute burn wound healing

**DOI:** 10.1371/journal.pone.0197744

**Published:** 2018-05-22

**Authors:** Yu-Wei Chang, Yi-Chia Wu, Shu-Hung Huang, Hui-Min David Wang, Yur-Ren Kuo, Su-Shin Lee

**Affiliations:** 1 Department of Post-Baccalaureate Medicine, Kaohsiung Medical University, Kaohsiung, Taiwan; 2 Division of Plastic Surgery, Department of Surgery, Kaohsiung Medical University Hospital, Kaohsiung, Taiwan; 3 Department of Plastic Surgery, Kaohsiung Municipal Ta-Tung Hospital, Kaohsiung, Taiwan; 4 Center for Stem Cell Research, Kaohsiung Medical University, Kaohsiung, Taiwan; 5 Ph. D. Program in Translational Medicine, Kaohsiung Medical University and Academia Sinica, Kaohsiung, Taiwan; 6 Department of Surgery, Faculty of Medicine, College of Medicine, Kaohsiung Medical University, Kaohsiung, Taiwan; 7 Orthopaedic Research Center, Kaohsiung Medical University, Kaohsiung, Taiwan; 8 Graduate Institute of Biomedical Engineering, National Chung Hsing University, Taichung, Taiwan; Massachusetts General Hospital, UNITED STATES

## Abstract

Adipose-derived stem cells (ADSCs) transplant has been reported to be a potential treatment for burn wounds. However, the effects of autogenicity and allogenicity of ADSCs on burn wound healing have not been investigated and the method for using ADSCs still needs to be established. This study compared the healing effects of autologous and allogenic ADSCs and determined an optimal method of using ADSCs to treat acute burn wounds. Experiments were performed in 20 male Wistar rats (weight, 176–250 g; age, 6–7 weeks). Two identical full-thickness burn wounds (radius, 4 mm) were created in each rat. ADSCs harvested from inguinal area and characterized by their high multipotency were injected into burn wounds in the original donor rats (autologous ADSCs group) or in other rats (allogenic ADSCs group). The injection site was either the wound center or the four corners 0.5 cm from the wound edge. The reduction of burn surface areas in the two experimental groups and in control group were evaluated with Image J software for 15 days post-wounding to determine the wound healing rates. Wound healing was significantly faster in the autologous ADSCs group compared to both the allogenic ADSCs group (*p<0*.*05*) and control group (*p<0*.*05*). Wound healing in the allogenic ADSC group did not significantly differ from that in control group. Notably, ADSC injections 0.5cm from the wound edge showed significantly improved healing compared to ADSCs injections in the wound center (*p<0*.*05*). This study demonstrated the therapeutic efficacy of ADSCs in treating acute burn wounds in rats. However, only autologous ADSCs improved healing in acute burn wounds; allogenic ADSCs did not. This study further determined a superior location of using ADSCs injections to treat burn wounds including the injection site. Future studies will replicate the experiment in a larger and long-term scale burn wounds in higher mammalian models to facilitate ADSCs therapy in burn wound clinical practice.

## Introduction

According to the World Health Organization, burns cause an estimated 265,000 deaths worldwide each year, and non-fatal burn injury is the leading cause of morbidity [1–2]. Effective wound management is a major goal of burn therapy because it is an important determinant in the survival and prognosis of patients with severe burns [3–4]. Thus, improved techniques for timely and permanent closure of full-thickness burn wounds are urgently needed.

Currently available grafting techniques, including split-thickness grafts (STSG) and full-thickness grafts (FTSG), are well developed and are effective for improving appearance and function. However, the effectiveness of both methods are limited by the availability of donor skin in specific sites, particularly in patients with large total body surface area (TBSA) burns. Sophisticated skin grafting designs that have been developed to solve this problem include the “modified Meek technique” [5] and the “postage stamp autografting technique”[6]. In 2005, our team introduced the “flypaper technique,” which offered the same advantages of a nine-fold higher expansion rate like the postage stamp autografting technique but was performed with a simpler preparation procedure [7]. In 2007, our team further introduced the “right shift flypaper technique,” which reduces the distance between skin islands, reduces the time of wound healing, and improves the uniformity and regularity of epithelium growth [8]. However, a continuing problem is the limited availability of autologous donor skin. More beneficial approaches to manage burn wound are needed to develop the therapy and adjuvant treatments.

Adipose-derived stem cells (ADSCs) are increasingly being considered as cell therapy in different diseases. For example, ter Huurne M et al. reported that a single intra-articular injection of ADSCs significantly decreased synovitis score and cartilage damage by suppressing activation of synovial macrophages [9]. Recently, a phase I trial (NCT01585857) of ADSCs in osteoarthritis based on the expansion and injection of autologous ADSCs in knees was completed in France and Germany. Patients treated with low-dose ADSCs experienced significant improvements in pain levels and function compared with baseline. Recent literature also investigated the promising therapeutic future of ADSCs on various wound healings and the healing mechanisms [10–12]. For example, Kim et al. showed that ADSCs promoted wound healing by interacting with dermal fibroblasts [13]. Collawn et al. demonstrated the effects of ADSCs on wound healing and tissue regeneration after a laser injury [14], and Riccobono et al. showed that autologous ADSCs enhanced wound healing in patients with cutaneous radiation syndrome [15]. Additionally, clinical trials of ADSCs therapy for diabetic foot ulcers (NCT02394886) and chronic wounds (NCT02092870) have entered phase 1 and phase 2, respectively.

There are few studies reporting the application of ADSCs to acute burn injuries in the literature and the effectiveness is still uncertain. In a thorough systematic review, Condé-Green et al. revealed that fat grafts and ADSCs have beneficial effects on acute burn wound healing, but also pointed out the lack of decisive evidence [16]. A mouse model in Loder et al. showed that ADSCs therapy improved burn wound healing in terms of wound area, wound depth, and apoptotic activity [17]. However, another study by Karimi et al. reported no significant improvement utilizing ADSCs treatment in acute burn wound healing (*p*>0.05) [18]. Therefore, decisive statistical data are needed to evaluate and clarify the effectiveness of ADSCs therapy for treating burn wounds.

The efficacy of using autologous or allogeneic ADSCs in burn wounds has not been investigated. Consequently, it is not known whether this is a key factor in the therapeutic effect of wound healing. In this present study, we built a rat model with full-thickness burn wounds and used Qtracker^®^(Invitrogen^™^, CA., U.S.A.) labeled- ADSCs to investigate the effects of ADSCs on acute burn wound healing and compared the healing effects of autologous and allogenic ADSCs. A therapeutic model was also established to compare the effects of different ADSCs injection sites. These results could make a contribution to the clinical applications of ADSCs therapy for burn wound management in the future.

## Materials and methods

### Animals

All animal experiments were in accordance with the National Health Research Institutes guidelines (http://lac.nhri.org.tw/) and were approved by the Institutional Animal Care and Use Committee (IACUC) of Kaohsiung Medical University (Protocol Number 97147). Twenty male Wistar rats weighing 176–250 g and 6–7 weeks old were used for the experiments (BioLANCO Taiwan Co., Ltd). During the experiments, all animals were kept in separate cages to prevent trauma from contact with other animals. For post operation infection control, antibiotics feeding with Cephalexin 20 mg/kg twice a day, and wound care once a day with normal saline and povidone-iodine ointment without gauze- covering were administrated for wound care during the experiments. Ibuprofen 1.5 mg/100 g was given orally for analgesia after the wounding procedures or the debridement during the experiments. Euthanasia was performed by using carbon dioxide inhalation after all experiments were complete. To decrease the suffering, the rats were euthanized in their home cage. The death of rats was confirmed by ascertaining cardiac and respiratory arrest.

### Experimental design

Each experimental group included three rats. Under anesthesia by intraperitoneal injection of Zoletil 50 (50 mg/kg; Virbac Taiwan Co, Ltd, Taiwan), two identical wounds were created in each rat. Therefore, six wounds were evaluated in each experimental group (Number of animals = 3; Number of samples = 6). Wounds were evaluated for 15 days. Qtracker^®^ labeled-ADSCs were used to trace the location of injected ADSCs in the rats. Wound biopsies were performed in the additional Auto 0.5cm group (Number of animals = 1; Number of samples = 2) and Allo 0.5cm group (Number of animals = 1; Number of samples = 2) (Auto Biopsy and Allo Biopsy, respectively). Table 1 shows that the experiments included 20 animals.

**Table 1:** Experimental design.

Group	Treatment	Purpose
**No Treatment**	Burn + Punch (n = 6)	Evaluate healing
**Control**	Burn + Punch + Injection of cell injection medium (K-NAC) at wound center (n = 6)	Evaluate healing
**Auto Center**	Burn + Punch + Injection of autologous ADSCs (5 x10^6^ cells/ml) at wound center (n = 6)	Evaluate healing
**Auto 0.5cm**	Burn + Punch + Injection of autologous ADSCs (5 x10^6^ cells/ml) at four corners, 0.5cm from wound edge (n = 6)	Evaluate healing
**Allo Center**	Burn + Punch + Injection of allogenic ADSCs (5 x10^6^ cells/ml) at wound center (n = 6)	Evaluate healing
**Allo 0.5cm**	Burn + Punch + Injection of allogenic ADSCs (5 x10^6^ cells/ml) at four corners, 0.5cm from wound edge (n = 6)	Evaluate healing
**Auto Biopsy**	Burn + Punch + Injection of autologous ADSCs (5 x10^6^ cells/ml) at four corners, 0.5cm from wound edge (n = 2, two wounds per rat)	Biopsy (day 7)
**Allo Biopsy**	Burn + Punch + Injection of allogenic ADSCs (5 x10^6^ cells/ml) at four corners, 0.5cm from wound edge (n = 2, two wounds per rat)	Biopsy (day 5)

Auto Center = autologous center; Auto 0.5cm = autologous 0.5 cm; Allo Center = allogenic center;

Allo 0.5cm = allogenic 0.5cm; Auto Biopsy = autologous biopsy; Allo Biopsy = allogenic biopsy.

### Fat tissue harvesting and ADSCs cultivation

The ADSCs were prepared as described in Sheen et al [19]. Briefly, each rat was anesthetized by intraperitoneal injection of Zoletil 50 (50 mg/kg) and the subcutaneous fatty tissue (approximately 3 g) was resected from the left and right inguinal area. After the operation, antibiotics feeding with Keflex 20mg/kg twice day, and wound care once a day with normal saline and povidone-iodine ointment without gauze- covering were administrated for wound care. Ibuprofen 1.5mg/100g was given orally for analgesia after the operation. The adipose tissue was then washed with Dulbecco’s phosphate-buffered saline (DPBS) to remove erythrocytes. After centrifugation at 1500 rpm for 5 minutes, the supernatant containing fat tissue was transferred to another tube and washed. The tissue was then distributed into other tubes and incubated in Dulbecco’s modified Eagle medium (DMEM) containing 1 mg/mL collagenase, 2 mM n-acetylcysteine, and 0.2 mM ascorbic acid 2-phosphate at 37°C for 3 hours. The collagenase solution was then removed from the tissue by centrifugation at 1500 rpm for 5 minutes. The cell pellets were washed and incubated in DMEM with 10% fetal bovine serum (FBS), 2 mM N-acetyl-L-cysteine (NAC), and 0.2 mM L-ascorbic acid 2-phosphate in 5% CO_2_. After 24 hours incubation, unattached cells were removed by washing with Dulbecco’s Phosphate-Buffered Saline (DPBS). The cell culture medium for putative ADSCs, referred to as K-NAC medium, was a modified MCDB 153 (Invitrogen-Gibco) supplemented with 2mM NAC and 0.2mM L-ascorbic acid 2-phosphate. Next, 5 mL of K-NAC medium containing 5% FBS were added to each 25-cm^2^ flask, and the medium was changed every other day until confluence. These putative ADSCs were then collected for use by trypsinization and kept for subculturing or stored in liquid nitrogen.

### Multilineage differentiation of ADSCs: Adipogenesis, osteogenesis, and chodrogenesis

Putative ADSCs were differentiated into adipocytes, osteoblasts, and chondrocytes as described by Sheen et al [19]. In brief, cells initially propagated in K-NAC medium with 5% FBS were treated by different supplementations in DMEM [20–21] followed by the different induction procedures reported by Sheen et al and Lin et al [20, 22].

### Fluorescence labeling of ADSCs with Qtracker^®^

Stem cells used for wound healing were first labeled by *in vitro* fluorescent labeling. Cells were prepared for fluorescent labeling as follows. First, confluent cells from the first passage were lifted by treatment with trypsin-EDTA (GIBCO, Life Technologies Inc., Grand Island, NY) at 37°C for 5 minutes. The trypsin was inactivated with serum-containing medium, and the cell suspension was centrifuged at 1500 rpm for 5 minutes. The cells were then fluorescently labeled with Qtracker^®^(Invitrogen^™^) by Invitrogen^™^ protocol and the final concentration was quantified at 5 x10^6^ cells/ mL by a Countess^™^ Automated Cell Counter (Invitrogen^™^).

### Injection of ADSCs after induction and debridement of contact burn wounds

**Fig 1:**
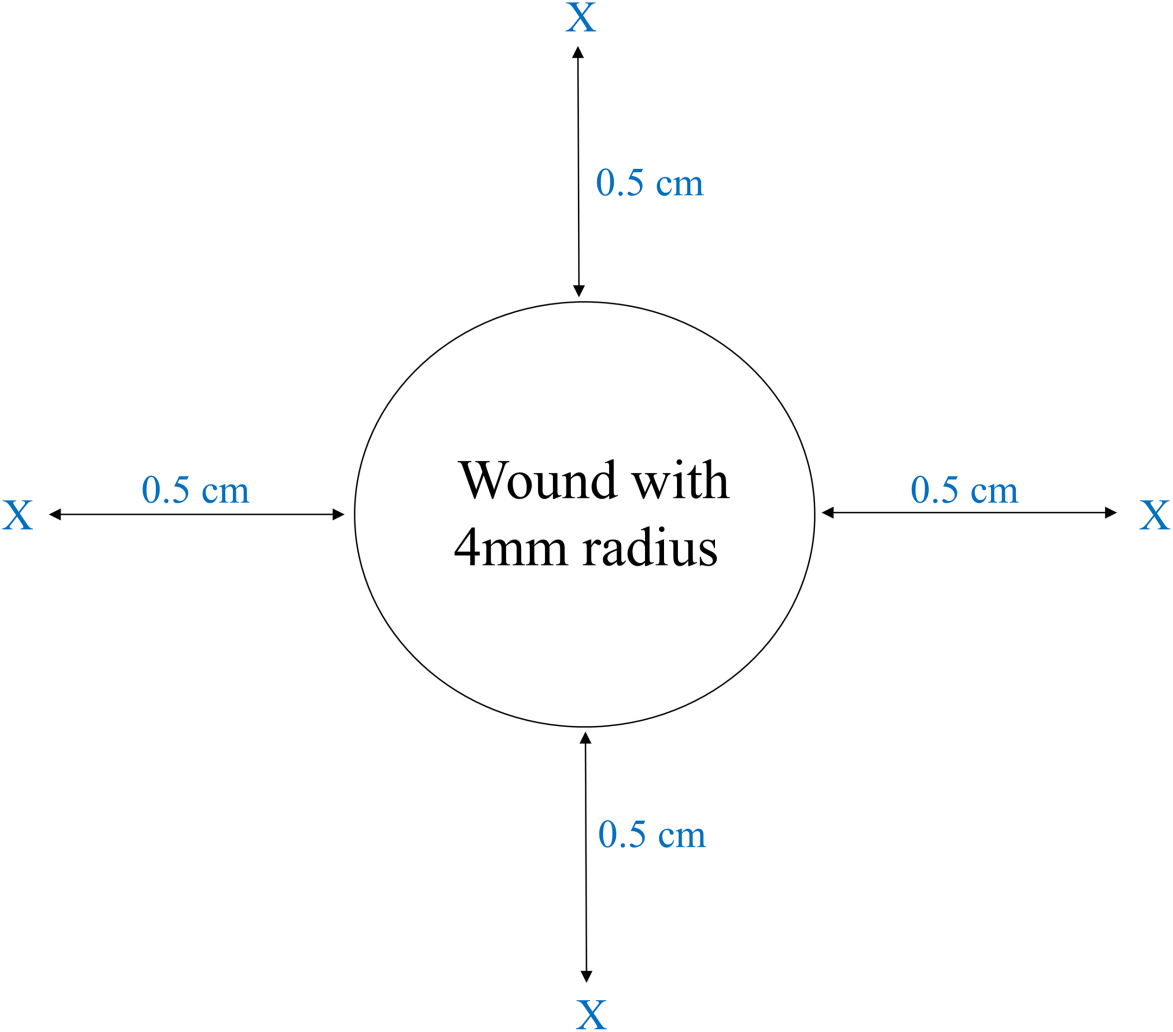
Injection of ADSCs at burn wound site. The autologous rat ADSCs or allogenic rat ADSCs (1 mL; 5 x10^6^ cells) were injected directly into the wound centers or into the four corners 0.5 cm from the wound edge (indicated by X). Each corner was injected with 250 μL of ADSCs.

Criteria including complete wound healing, normal activity without disability, and good appetite of rats were used to determine that rats had recovered from fat tissue harvesting. After total recovery from fat tissue resection in all rats, two identical, circular (radius, 4 mm), and full-thickness burn wounds were then created in the back of each rat with a metal rod (radius, 4 mm) heated to 95°C under the anesthesia by intraperitoneal injection of Zoletil 50 (50 mg/kg). The wounds were not sutured nor covered with any dressing. After 24 hours, debridement of the burn wounds was performed by using a punch (radius, 4 mm; *Miltex*^®^, NJ, USA) to cut to the level of panniculus carnosus under the anesthesia by intraperitoneal injection of Zoletil 50 (50 mg/kg). ADSCs were suspended in K-NAC medium (5 x 10^6^ cells/ml) for injection. According to a report from Childhood Burn Fundation of the Republic of China, the injection dosage with 5 x10^6^ ADSCs was effective to improve wound healing with a 4 mm radius. Therefore, we chose 5 x10^6^ cells of ADSCs as the injection dosage. Right after the debridement, autologous ADSCs, or allogenic ADSCs (5 x10^6^ cells) from a different rat, were injected into the center of the wound under the level of panniculus carnosus (Auto Center group and Allo Center group) or at the four corners 0.5 cm from the edge of the wound to the subcutaneous tissue (Auto 0.5 cm group and Allo 0.5 cm group) (Fig 1) under the anesthesia by intraperitoneal injection of Zoletil 50 (50 mg/kg). The two other groups in the experiments were a No Treatment group (burn and punch only) and a Control group (burn, punch, and injection of K-NAC medium at the wound center) (Table 1).

### Burn wound size measurement and analysis

In each group of three animals, wound sizes were measured three times a day on days 1, 2, 5, 7, 9, 11, 13, and 15. In each animal, the wounds were digitally photographed at the indicated time intervals. Camera was fixed on a tripod to standardize the length item and the lighting setting was the same in each shot. A ruler was put beside the wound as a comparable scale. Rats were anesthetized with isoflurane 1% and photographed under the same posture. The wound areas were automatically calibrated and calculated by Image J software (National Institutes of Health, NIH http://rsb.info.nih.gov/ij). The wound areas were standardized by comparing with the original wound size and expressed as a percentage of wound closure as follows: [(day 0 area − day n area) /(day 0 area)] x100% [23].

### Histology and immunofluorescence studies of specimens

The survival and the resident of ADSCs in burn wound area were comfirmed. Skin covering the margin of unwounded skin and the ADSCs injection site (i.e. 0.5 cm from the wound margin) were punched and collected by using a 4-mm radius punch (Miltex^®^) in the group of autologous biopsy (Auto Biopsy) and allogenic biopsy (Allo Biopsy) on day 7 and day 5, respectively. Each specimen was fixed in Histochoice for histopathological and biopsy analysis [23]. Immediately after excision, tissue samples were rinsed in isotonic saline and then fixed in 4% paraformaldehyde for 24 hours. After fixation, tissues were cryopreserved in an isotonic 30% sucrose solution for at least 24 hours. Qtracker^®^ labeled- ADSCs injected on day 1 were used for stem cell tracking [20].

### Statistical analysis

Paired t-test analysis was used to make pairwise comparisons between the groups (n = 6). A *p* value < 0.05 was considered statistically significant.

## Results

### Characterization and verification of rat ADSCs

**Fig 2:**
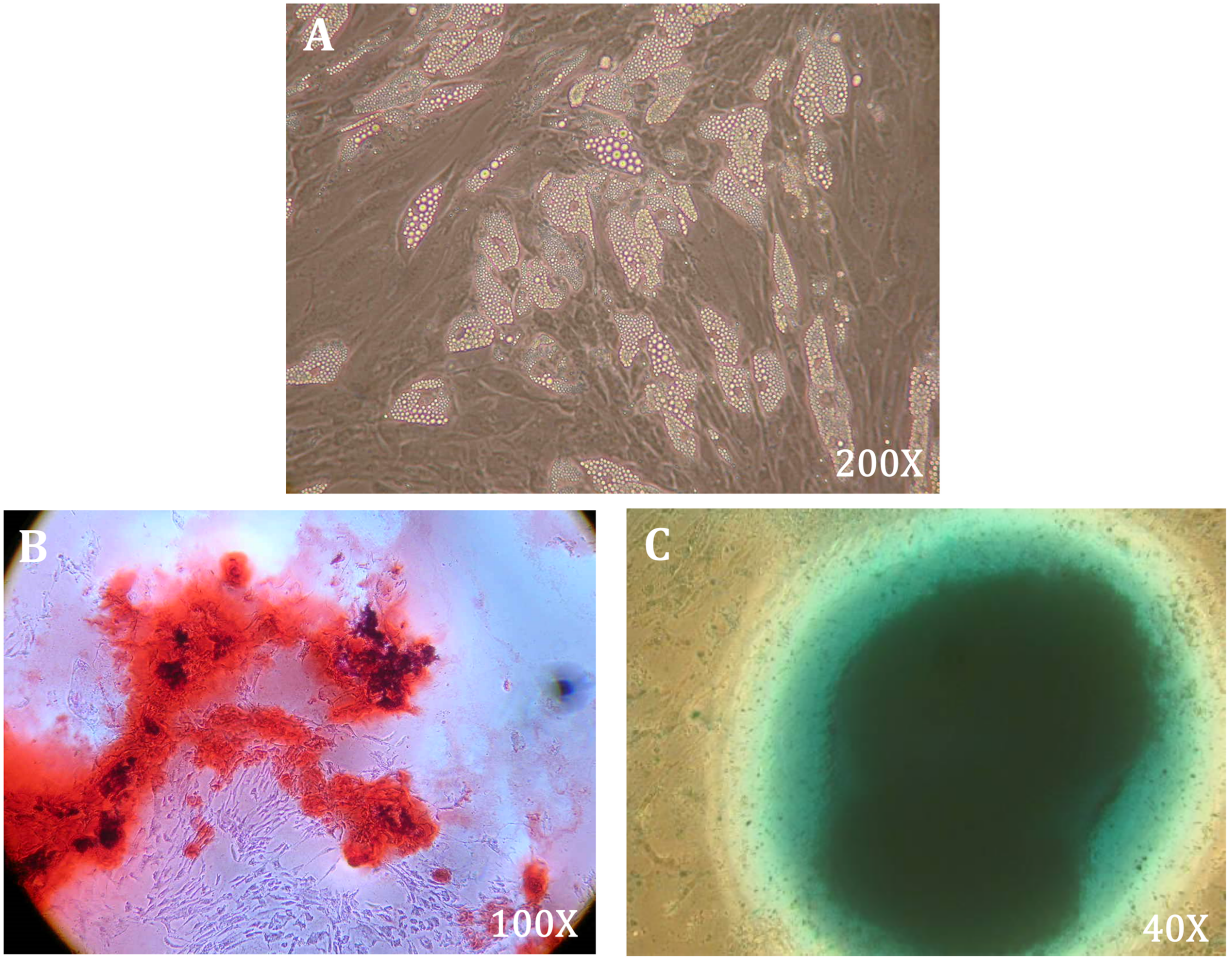
Characterization of multilineage differentiation capability of rat ADSCs. The multilineage differentiation capability of cultured ADSCs were characterized by induction of multilineage cell differentiation, including (A) adipogenesis, 200X magnification; (B) osteogenesis, Alizarin red staining, 100X magnification; and (C) chondrogenesis, Alcian Blue staining, 40X magnification.

To analyze multilineage differentiation and characterize the putative ADSCs, this study experimentally induced adipogenesis, osteogenesis, and chondrogenesis of the harvested cells (Fig 2A–2C, respectively). The identities of putative rat ADSCs up to three passes were also confirmed by fluorescence-activated cell sorting analysis as described in our previous works [19]. The ADSCs were positive for surface markers of CD90, CD34, and CD29, and negative for surface markers of CD45 and CD31.

### Full-thickness contact burn wound

**Fig 3:**
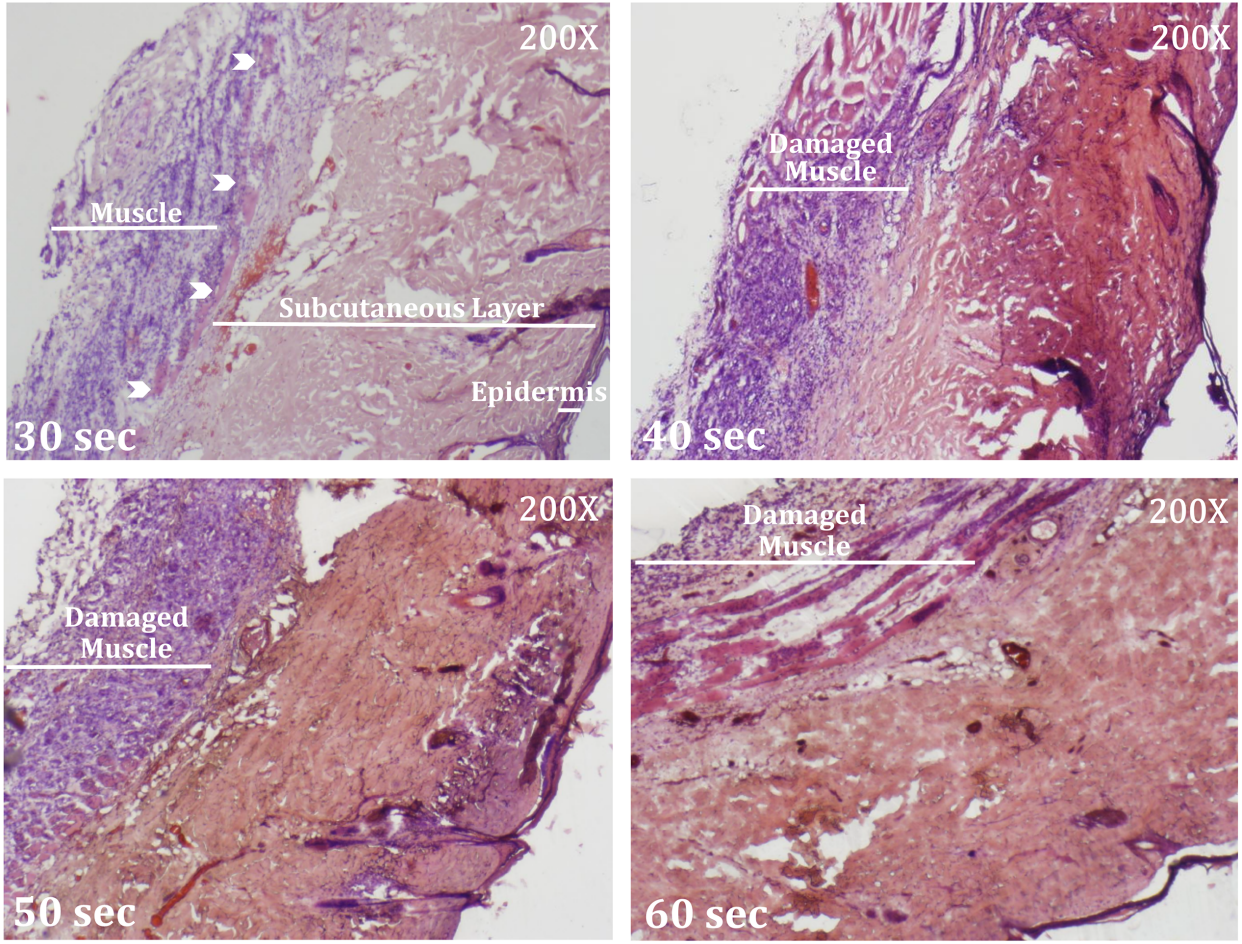
Full-thickness contact burn wound. The H&E staining results for burn wounds created by varying durations of contact (30, 40, 50, and 60 seconds) with a heated metal rod. In the 30 second group, the full-thickness of the skin was burned but the muscle layer was intact. White arrow indicates the panniculus carnosus. Note the damaged muscle layers (inflammation in 40 and 50 seconds groups; necrosis in 60 seconds group) in the wounds created by contact longer than 30 seconds.

Burn wounds were experimentally created in rats through varying durations of contact with a heated metal rod (30, 40, 50, and 60 seconds; 95°C). The results showed that contact for 40 and 50 seconds partially damaged the muscle layer, whereas contact for 60 seconds damaged the full muscle layer. Contact for 30 seconds burned the full-thickness of the skin without damaging the muscle layer (Fig 3). Therefore, the following experiments evaluated the effects of ADSCs injections in burn wounds created by 30 seconds of contact with the skin.

### Injected ADSCs remained viable and targeted the burn wounds

**Fig 4:**
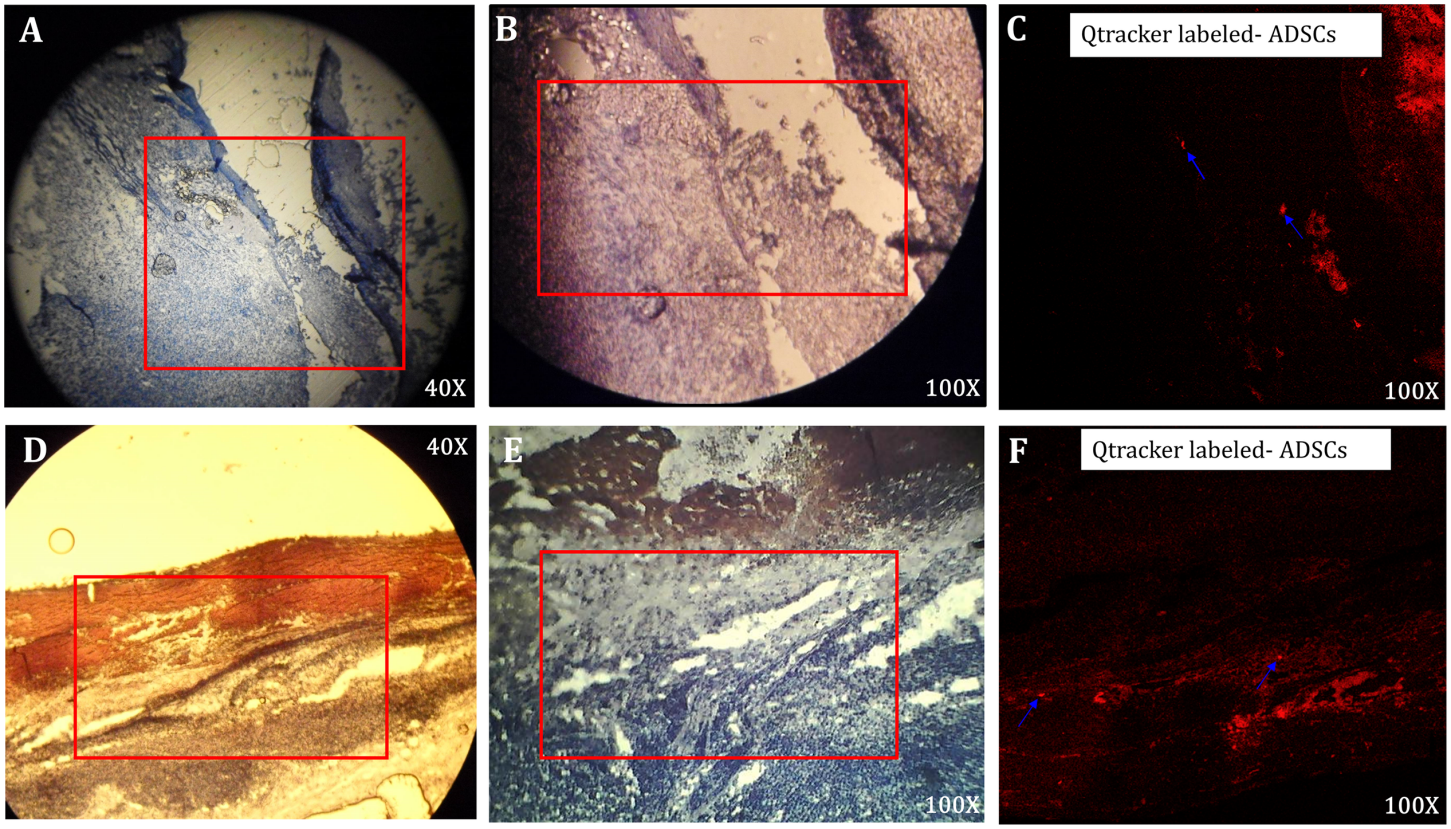
Localization of ADSCs in wound by Qtracker^®^ labeled- ADSCs. (A-C) The H&E staining results 7 days after injection of autologous ADSCs at the four corners, 0.5cm from the wound edge. (A) 40X magnification, (B) 100X magnification, and (C) visualization by Qtracker^®^ labeling under 100X magnification. (D-F) The H&E staining results 5 days after injection of allogenic ADSCs at the four corners, 0.5cm from the wound edge. (D) 40X magnification, (E) 100X magnification, and (F) visualization by Qtracker^®^ labeling under 100X magnification. The Qtracker^®^ labeled- ADSCs are indicated by blue arrows.

To determine whether the injected ADSCs remained viable and targeted burn wounds through the early stage of wound healing, Qtracker^®^ labeled- ADSCs were used to track the localization of ADSCs injected at different time points. Fig 4 shows that both the autologous and allogenic ADSCs injected 0.5 cm from the edge of the wound were viable and localized in the wound on day 5 and day 7, respectively (Fig 4).

### Autologous ADSCs facilitated acute burn wound healing but allogenic ADSCs did not

Burn wound healing was then evalauted by calculating the reduction of burn surface area, which stood for the wound healing rate. Although the wounds in all groups appeared similar in gross view, wound healing rates differed. In the experiment group, the ADSCs were cultured with the medium K-NAC. Therefore, we used the medium K-NAC as the injection medium in the control group. Experimental applications of injecting fresh medium K-NAC in the control group showed a significanlty higher wound healing rate compared to the No Treatment group (*p*<0.05) (Table 2). Thus, the group injected with fresh medium K-NAC was designated as the appropriate control group in subsequent experiments.

**Table 2:** Wound healing rate of no treatment VS control.

Healing Rate%	Day 2	Day 5	Day 7	Day 9	Day 11	Day 13	Day 15
**No Treatment**	17.19±0.93	26.77±1.48	40.16±1.30	56.62±1.62	67.45±1.92	77.86±1.90	85.13±1.85
Control*	14.25±1.03	29.60±1.83	43.52±1.05	60.02±1.38	73.00±1.16	82.67±1.17	91.41±0.41

Mean ±S.E.M; *p* values were calculated by *paired t- test*.

Number of animals = 3; Number of samples = 6.

*Significant difference from No Treatment (p<0.05)

**Fig 5:**
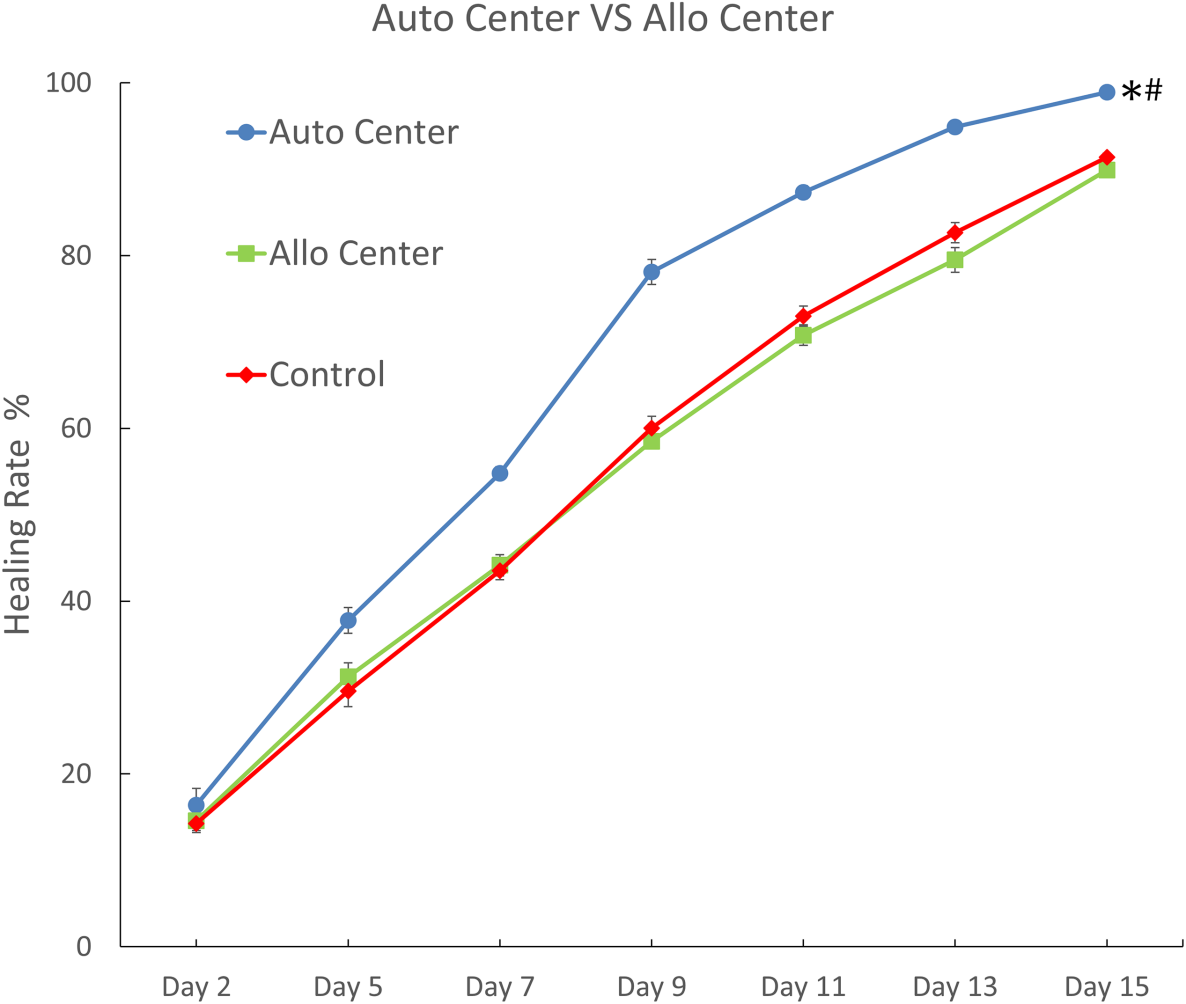
Comparison of burn wound healing rates between autologous and allogenic ADSCs treatment. Burn wound healing rates on different days were calculated for the group treated by injection of autologous ADSCs at the wound center (Auto Center), the group treated by injection of allogenic ADSCs at the wound center (Allo Center), and the group treated by injection of K-NAC medium at the wound center (Control.) Data are shown as mean ± S.E.M. (Number of animals = 3; Number of samples = 6). *Significant difference from Control (*p*<0.05). ^#^Significant difference from Allo Center (*p*<0.05). *p* values were calculated by *paired t- test*.

Further comparisons showed that the Auto Center group, which had autologous ADSCs injected in the wound centers, displayed significantly higher wound healing rates compared to the control group at each time point through day 15 (*p*<0.05; Fig 5 and S1 Table) Additionally, wound healing in the Auto Center group was 99% complete by day 15. The healing efficacy in the Auto Center group was also significantly higher than that in the Allo Center group, which had allogenic ADSCs injected in the wound centers (*p* < 0.05; Fig 5) However, allogenic and autologous ADSCs had completely different healing effects. Healing efficacy did not significantly differ between the Allo Center group and the control group (Fig 5). In summary, healing of acute burn wounds was enhanced by autologous ADSCs, but not by allogenic ADSCs.

### Injection of ADSCs 0.5cm from wound edge enhances burn wound healing

**Fig 6:**
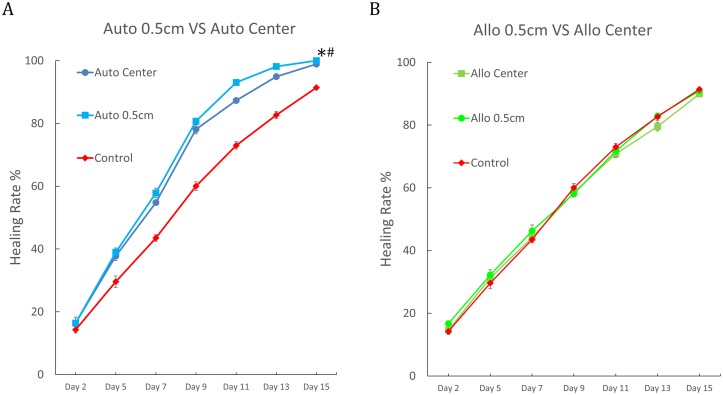
Comparison of burn wound healing rates between different ADSC injection sites. (A) Burn wound healing rates on different days in the group treated by injection of autologous ADSCs at the wound center (Auto Center), the group treated by injection of autologous ADSCs in the four corners 0.5cm from the edge of the wound (Auto 0.5cm), and the group treated by injection of K-NAC medium at the wound center (Control). Data are shown as mean ± S.E.M. (Number of animals = 3; Number of samples = 6). *Significant difference of Auto 0.5cm from Control (*p*<0.05). ^#^Significant difference of Auto 0.5cm from Auto Center (*p*<0.05). *p* values were calculated by *paired t- test*. (B) Burn wound healing rates on different days in the group treated by injection of allogenic ADSCs at the wound center (Allo Center), the group treated by injection of allogenic ADSCs in the four corners 0.5cm from the edge of the wound (Allo 0.5cm), and the group treated by injection of K-NAC medium at the wound center (Control.) Data are shown as mean ±S.E.M. (Number of animals = 3; Number of samples = 6).

To develop potential clinical applications of the proposed ADSCs therapy, further experiments were performed to determine an optimal site of ADSCs injection. The results showed that compared to the Auto Center group, wound healing was significantly faster in the Auto 0.5cm group, where autologous ADSCs were injected around 0.5cm from the wound edge (*p* < 0.05; Fig 6A) Moreover, the wounds in the Auto 0.5cm group were 100% healed by day 15 (S2 Table) Although the Allo 0.5cm group showed significantly faster wound healing compared to the Allo Center group (*p* < 0.05; S2 Table), neither group showed significantly better healing compared to the control group (Fig 6B) Only autologous ADSCs improved the rate of acute burn wound healing regardless of injection site. Allogenic ADSCs did not improve healing at all.

## Discussion

In this present study, we explored the impact of ADSCs on acute burn wound healing.

First, the ADSCs isolated from rats were characterized by their multi-lineage differentiation capability, and the clinical picture of burn wound management was simulated by creating a full-thickness burn wound followed by the debridement of the wound. After the early period of burn wound healing, cell viability and the resident of stem cells injected into the burn wounds were confirmed by Qtracker^®^ labeling. This study is the first to report that treatment with autologous ADSCs significantly reduced the burn wound area, and the treatment with allogenic ADSCs did not. Moreover, comparisons of different ADSCs injection sites showed that an optimal injection site in terms of wound healing was 0.5 cm from the edge of the wound.

Our quantitative analyses demonstrated the therapeutic efficacy of ADSCs injections for treating acute burn wounds, which is consistent with Loder et al. (2015), but not with Karimi et al. (2014) [17–18]. To explain the different outcomes reported in the literature, this study evaluated two important factors: the effect of ADSCs injection medium on wound healing and the autogenicity / allogenecity of the ADSCs.

First, the effect of the cell injection medium K-NAC was investigated. During burn wound surgeries, the surgeon may consider no treatment as the baseline of stem cell treatment. For example, the control group in the series of Karimi was untreated mice [18]. However, when performing stem cell therapy, the application of medium is necessary nowadays. Our experiments showed that the medium itself possessed a healing effect on burn wounds, and the healing effect was significantly larger than that in the “No Treatment group” (Table 2) (*p* = 0.0138). The therapeutic effect of the medium may result from its components which include amino acids, Vitamin B-12, and Vitamin C, etc. In addition, medium may also provide the moist effect which is beneficial for wound healing. Consequently, using cell injection medium (K-NAC medium) as the “control group” is important in clarifying the therapeutic efficacy of the stem cells.

Second, the effects of autogenicity and allogenicity on the healing effects of ADSCs was elucidated. The C567BL/6 and Balb/c mice used in Loder et al [17] and Karimi et al [18], respectively, were from the same inbreeding descendant. However, the ADSCs were harvested from different individuals. Thus, the ADSCs used in both studies (Loder et al [17] and Karimi et al [18]) were regarded as syngenic ADSCs but not autogenic ADSCs. In clinical scenario, the use of syngenic cell for human cell therapy is rare. Our outbreed Wistar rats in this study provided a more clinically relevant model of autologous and allogenic ADSCs transplantation.

This study is the first to report that the treatment with autologous ADSCs significantly reduced the burn wound area, but the treatment with allogenic ADSCs did not. Our results suggests that the discrepancies among previous conflicting studies regarding the healing efficacy of ADSCs was the result of the autogenicity / allogenicity of the ADSCs. Various response involved in ADSCs wound healing therapy has been reported including changes in anti-inflammatory cytokine expression, suppression of T-cell proliferation, increased regulation of T cell expression, and direct differentiation of ADSCs themselves [11,24]. We still don’t know why the allogenic ADSCs did not improved the healing. Compared to autologous ADSCs, allogenic ADSCs have more risk to initiate immune response during therapy. Since a thermal injury itself substantially increases production of inflammatory mediators such as cytokines and chemokines during the healing process [4,25], the subsequent immune reaction to the injection of ADSCs might have interfered with immune signal transduction and the wound healing process. In the present study, allogenic ADSCs did not improved burn wound healing. The immune response induced by allogenic ADSCs may play a role in that. However, more studies are needed to clarify that. In addition, there are some risks have been reported for applying allogenic stem cell therapy in other disease. Long-term side effects from infections related to abnormal immune reconstitution and secondary cancers may appear after allogenic hematopoietic stem cell transplantation [26]. An allogenic stem cell transplant for acute myeloid leukemia may cause not only graft-versus-host disease but also mucositis [27–28]. It seems that applying autologous ADSCs therapy is beneficial.

The ADSCs dosage and injection sites are also important factors in the therapeutic efficacy of ADSCs therapy [18]. The present study shown that 5 x10^6^ cells was a sufficient quantity for ADSCs therapy in acute burn wounds with 4mm radius and the injection to the 0.5cm from the edge was more appropriate than the center of the wound. Compared to the burn wound area and the surrounding area, cell proliferation, differentiation, and vascularization are generally destroyed in burn wound area [29]. Therefore, the responses of ADSCs in wound center and wound edge tissue should be different. Further studies are needed to optimize the clinical applications of ADSCs in burn wound therapy.

Many novel uses of ADSCs for wound healing have been reported. For example, Nambu et al. (2007) showed that healing-impaired wounds treated with ADSC containing-atelocollagen matrix with silicone membrane (ACMS) have advanced granulation tissue and capillary formation [30]. Orbay et al. showed that an ADSCs-seeded acellular dermal matrix (ADM) provided a better implant scaffold in terms of wound vascularization, volume maintenance, and collagen quantity [31]. For nonhealing wounds, Lafosse et al. designed a biological dressing made of autologous ADSCs and absorbable human cellular collagen matrix. Long-term follow up showed that the dressing effectively improved tissue remodeling and angiogenic dynamics [32]. Accordingly, ADSCs itself not only has great potential by direct injection of ADSCs into the wound, but also plays a key role in wound dressing design. Furthermore, our study addressing the importance of autogenicity of ADSCs is crucial for the development of biological dressings containing ADSCs for burn wounds in the future.

Recently, stem cells have been applied to promote superior healing of the wounds. Not only stem cells have been shown to promote better and faster healing of the burn wounds, but also they have decreased the inflammation levels with less scar progression and fibrosis [33]. In some special circumstances, using ADSCs to treat the wounds with small size has great advantages. First, due to its noninvasive characteristic, ADSCs is suitable to treat the wound with difficult healing, for example, diabetic mellitus foots [34 and NCT02394886]. Second, in the wound and the burn wound in critical or complex anatomical areas like facial area or ear, ADSCs treatment might be a good alternative way other than skin graft. However, a lot of related techniques still need to be developed for replacing graft with ADSCs therapy. Our present study showed the therapeutic efficacy of ADSCs in the wound with small size still gives precious background information for developing related clinical application in the future.

There are several limitations in the present study. The first limitation is the size of the burn wounds. The experimentally created burn wounds were relatively small (4 mm radius) whereas fire or scald wounds reported worldwide have an average TBSA of approximately 20% [35]. Since the immune environment may substantially differ between large and small burn wounds, future studies of larger burn wounds that are better representations of the typical clinical scenario are needed to further evaluate the therapuetic efficacy of ADSCs in clinical practice.

The second limitation is the use of the rat model for studying human wound healing. As the histological structures are different in human and rats, the healing characteristics have some differences between them. Contraction plays an important role in wound healing in rats. However, the wounds of human mainly heal by re-epithelialization. Although the panniculus carnosus which plays an important role in wound contraction in rats has been removed after debridement, the involvement of contraction did not completely exclude in the present study. Although the rat model does not completely stand for the wound healing in humans, rats studies still contributed to develop the related therapy in clinical [17, 24, 34]. The present study gives important information of the ADSCs treatment to burn wound healing. Future experiments of pig model will be performed to further evaluate the therapeutic efficacy of ADSCs in human wound healing.

Additionally, a noted limitation of the proposed therapy is the large quantity of ADSCs required for injection into the wounds, i.e. 5 x10^6^ cells. Even larger quantities of ADSCs would be needed for ADSCs therapy in major burn patients. Thus, *in vitro* cell expansion would be required for ADSCs therapy in major burn patients and the carcinogenic risk reported by Herberts et al. and Schweizer et al [36,37] would increase accordingly. This carcinogenic phenomenon was also observed in human embryonic stem cells and human mesenchymal stem cells, respectively [38,39].

Finally, although the oncogenic safety of biological dressings composed of autologous ADSCs (up to passage 4) and a cellular collagen matrix has been confirmed in terms of genetic stability and tumorigenicity in rat and human studies [32], further large-scale *in vitro* and *in vivo* studies of a longer cell passage are needed to identify long-term adverse effects and evaluate the carcinogenic risk of ADSCs therapy for burn wounds.

## Conclusion

In conclusion, quantitative analysis in this study demonstrated the therapeutic efficacy of using ADSCs isolated from the rat inguinal area to treat acute burn wounds. Our experiments showed that only autologous ADSCs enhanced burn wound healing; allogenic ADSCs did not. The proposed ADSCs therapy for burn wound healing was further optimized in terms of injection dosage and injection site. Our future research will aim to optimize the methodology of using ADSCs in clinical applications and evaluate its long-term pre-clinical outcomes in hugher mammalian model.

## Supporting information

S1 Table Wound healing rate of autologous ADSC VS allogenic ADSC. (DOCX)

S2 Table Wound healing rate of injection at center VS injection at 0.5cm from wound edge. (DOCX)

## References

[1] World Health Organization. 2016 Burns Fact Sheet worldwide. 2017; 2:9. http://www.who.int/mediacentre/factsheets/fs365/en/

[2] Peck MD. Epidemiology of burns throughout the world. Part I: Distribution and risk factors. Burns. 2011; 37(7): 1087–1100. doi: 10.1016/j.burns.2011.06.005 21802856 10.1016/j.burns.2011.06.005

[3] Church D, Elsayed S, Reid O, Winston B, Lindsay R. Burn Wound Infections. Clin Microbiol Rev. 2006; 19(2): 403–434. doi: 10.1128/CMR.19.2.403-434.2006 16614255 10.1128/CMR.19.2.403-434.2006 PMC1471990

[4] Rowan MP, Cancio LC, Elster EA, Burmeister DM, Rose LF, Natesan S, et al Burn wound healing and treatment: review and advancements. Critical Care. 2015; 19(1). 10.1186/s13054-015-0961-2 PMC4464872 26067660

[5] Kreis RW, Mackie DP, Vloemans AW, Hermans RP, Hoekstra MJ. Widely expanded postage stamp skin grafts using a modified Meek technique in combination with an allograft overlay. Burns. 1993; 19(2): 142–145. 8471149 10.1016/0305-4179(93)90038-a

[6] Lee SS, Tsai CC, Lai CS, Lin SD. An easy method for preparation of postage stamp autografts. Burns. 2000; 26(8): 741–749. 11024609 10.1016/s0305-4179(00)00050-4

[7] Lee SS, Lin TM, Chen YH, Lin SD, Lai CS. “Flypaper technique” a modified expansion method for preparation of postage stamp autografts. Burns. 2005; 31(6): 753–757. 15955633 10.1016/j.burns.2005.04.001

[8] Lee SS, Chen YH, Sun IF, Chen MC, Lin SD, Lai CS. “Shift to right flypaper technique” a refined method for postage stamp autografting preparation. Burns. 2007; 33(6): 764–769. doi: 10.1016/j.burns.2006.10.383 17524561 10.1016/j.burns.2006.10.383

[9] ter Huurne M, Schelbergen R, Blattes R, Blom A, de Munter W, Grevers L, et al Antiinflammatory and chondroprotective effects of intraarticular injection of adipose-derived stem cells in experimental osteoarthritis. Arthritis Rheum 2012; 64(11): 3604–3613. doi: 10.1002/art.34626 22961401 10.1002/art.34626

[10] Kim WS, Park BS, Sung JH. The wound-healing and antioxidant effects of adipose-derived stem cells. Expert Opin Biol Ther. 2009; 9(7): 879–887. doi: 10.1517/14712590903039684 19522555 10.1517/14712590903039684

[11] Hassan WU, Greiser U, Wang W. Role of adipose-derived stem cells in wound healing. Wound Repair Regen. 2014; 22(3): 313–325. doi: 10.1111/wrr.12173 24844331 10.1111/wrr.12173

[12] Ojeh N, Pastar I, Tomic-Canic M, Stojadinovic O. Stem cells in skin regeneration, wound healing, and their clinical applications. Int J Mol Sci. 2015; 16(10): 25476–25501. doi: 10.3390/ijms161025476 26512657 10.3390/ijms161025476 PMC4632811

[13] Kim WS, Park BS, Sung JH, Yang JM, Park SB, Kwak SJ, et al Wound healing effect of adipose-derived stem cells: A critical role of secretory factors on human dermal fibroblasts. J Dermatol Sci. 2007; 48(1): 15–24. doi: 10.1016/j.jdermsci.2007.05.018 17643966 10.1016/j.jdermsci.2007.05.018

[14] Collawn SS, Banerjee NS, de la Torre J, Vasconez L, Chow LT. Adipose-derived stromal cells accelerate wound healing in an organotypic raft culture model. Ann Plast Surg. 2012; 68(5): 501–504. doi: 10.1097/SAP.0b013e31823b69fc 22510896 10.1097/SAP.0b013e31823b69fc PMC3477580

[15] Riccobono D, Agay D, Scherthan H, Forcheron F, Vivier M, Ballester B et al Application of adipocyte-derived stem cells in treatment of cutaneous radiation syndrome. Health Phys. 2012; 103(2): 120–126. doi: 10.1097/HP.0b013e318240595b 22951469 10.1097/HP.0b013e318240595b

[16] Condé-Green A, Marano AA, Lee ES, Reisler T, Price LA, Milner SMet et al Fat grafting and adipose-derived regenerative cells in burn wound healing and scarring. Plast Reconstr Surg. 2016; 137(1): 302–312. doi: 10.1097/PRS.0000000000001918 26710034 10.1097/PRS.0000000000001918

[17] Loder S, Peterson JR, Agarwal S, Eboda O, Brownley C, DeLaRosa S, et al Wound healing after thermal injury is improved by fat and adipose-derived stem cell isografts. J Burn Care Res. 2015; 36(1): 70–76. doi: 10.1097/BCR.0000000000000160 25185931 10.1097/BCR.0000000000000160 PMC4286508

[18] Karimi H, Soudmand A, Orouji Z, Taghiabadi E, Mousavi SJ. Burn wound healing with injection of adipose-derived stem cells: a mouse model study. Ann Burns Fire Disasters. 2014; 27(1): 44–9. 25249847 PMC4158446

[19] Sheen YT, Lin TM, Chang KP, Lai CS, Lin SD, Lee SS. Commercially available materials as scaffold candidates for adipose-derived stromal/progenitor cell tissue engineering. Formosan Journal of Surgery. 2014; 47(1): 1–10.

[20] Zuk PA, Zhu M, Ashjian P, De Ugarte DA, Huang JI, Mizuno H et al Human Adipose Tissue Is a Source of Multipotent Stem Cells. Mol Biol Cell. 2002; 13(12): 4279–4295. doi: 10.1091/mbc.E02-02-0105 12475952 10.1091/mbc.E02-02-0105 PMC138633

[21] Pittenger MF, Mackay AM, Beck SC, Jaiswal RK, Douglas R, Mosca JD, et al Multilineage Potential of Adult Human Mesenchymal Stem Cells. Science. 1999; 284(5411): 143–147. 10102814 10.1126/science.284.5411.143

[22] Lin TM, Chang HW, Wang KH, Kao AP, Chang CC, Wen CH, et al Isolation and identification of mesenchymal stem cells from human lipoma tissue. Biochem Biophys Res Commun. 2007; 361(4): 883–889. doi: 10.1016/j.bbrc.2007.07.116 17679141 10.1016/j.bbrc.2007.07.116

[23] Chunmeng S, Tianmin C, Yongping S, Xinze R, Yue M, Jifu Q, et al Effects of dermal multipotent cell transplantation on skin wound healing. J Surg Research. 2004; 121(1): 13–19. 15313369 10.1016/j.jss.2004.04.008

[24] Kuo YR, Chen CC, Goto S, Lee IT, Huang CW, Tsai CC, et al Modulation of Immune Response and T-Cell Regulation by Donor Adipose-Derived Stem Cells in a Rodent Hind-Limb Allotransplant Model. Plast Reconstr Surg. 2011; 128(6): 661e–672e. doi: 10.1097/PRS.0b013e318230c60b 22094768 10.1097/PRS.0b013e318230c60b

[25] Rose LF, Chan RK. The Burn Wound Microenvironment. Advances in Wound Care. 2016; 5(3): 106–118. doi: 10.1089/wound.2014.0536 26989577 10.1089/wound.2014.0536 PMC4779284

[26] Mohty B and Mohty M. Long-term complications and side effects after allogeneic hematopoietic stem cell transplantation: an update. Blood Cancer J. 2011;1(4): e16 doi: 10.1038/bcj.2011.14 22829137 10.1038/bcj.2011.14 PMC3255242

[27] Bellm LA, Epstein JB, Rose-Ped A, Martin P, Fuchs HJ. Patient Reports of Complications of Bone Marrow Transplantation. Support Care Cancer. 2000; 8:33–39. 10650895 10.1007/s005209900095

[28] Lamarthée B, Malard F, Saas P, Mohty M, Gaugler B. Interleukin-22 in Graft-Versus-Host Disease after Allogeneic Stem Cell Transplantation. Front Immunol. 2016; 7: 148 doi: 10.3389/fimmu.2016.00148 27148267 10.3389/fimmu.2016.00148 PMC4836046

[29] Tiwari VK. Burn wound: How it differs from other wounds? Indian J Plast Surg. 2012; 45(2): 364–373. doi: 10.4103/0970-0358.101319 23162236 10.4103/0970-0358.101319 PMC3495387

[30] Nambu M, Ishihara M, Nakamura S, Mizuno H, Yanagibayashi S, Kanatani Y, et al Enhanced healing of mitomycin C-treated wounds in rats using inbred adipose tissue-derived stromal cells within an atelocollagen matrix. Wound Repair Regen. 2007; 15(4): 505–510. doi: 10.1111/j.1524-475X.2007.00258.x 17650094 10.1111/j.1524-475X.2007.00258.x

[31] Orbay H, Takami Y, Hyakusoku H, Mizuno H. Acellular Dermal Matrix Seeded with Adipose-Derived Stem Cells as a Subcutaneous Implant. Aesth Plast Surg. 2011; 35(5): 756–763. 10.1007/s00266-011-9683-2 21416297

[32] Lafosse A, Desmet C, Aouassar N, André W, Hanet M, Beauloye C, et al Autologous Adipose Stromal Cells Seeded onto a Human Collagen Matrix for Dermal Regeneration in Chronic Wounds. Plast Reconstr Surg. 2015; 136(2): 279–295. doi: 10.1097/PRS.0000000000001437 25946602 10.1097/PRS.0000000000001437

[33] Ghieh F, Jurjus R, Ibrahim A, Geagea AG, Daouk H, El Baba B, et al The Use of Stem Cells in Burn Wound Healing: A Review. Biomed Res Int. 2015; 2015:684084 doi: 10.1155/2015/684084 26236731 10.1155/2015/684084 PMC4508388

[34] Kuo YR, Wang CT, Cheng JT, Kao GS, Chiang YC, Wang CJ. Adipose-Derived Stem Cells Accelerate Diabetic Wound Healing Through the Induction of Autocrine and Paracrine Effects. Cell Transplant. 2016;25(1):71–81. doi: 10.3727/096368915X687921 25853951 10.3727/096368915X687921

[35] Peck MD. Epidemiology of burns throughout the World. Part II: Intentional burns in adults. Burns. 2012; 38(5): 630–637. doi: 10.1016/j.burns.2011.12.028 22325849 10.1016/j.burns.2011.12.028

[36] Herberts CA, Kwa MS, Hermsen HP. Risk factors in the development of stem cell therapy. J Transl Med. 2011; 9(1): 29. 21418664 10.1186/1479-5876-9-29 PMC3070641

[37] Schweizer R, Tsuji W, Gorantla VS, Marra KG, Rubin JP, Plock JA. The role of adipose-derived stem cells in breast cancer progression and metastasis. Stem Cells International. 2015; 2015: 1–17. 10.1155/2015/120949 PMC4427098 26000019

[38] Närvä E, Autio R, Rahkonen N, Kong L, Harrison N, Kitsberg D, et al High-resolution DNA analysis of human embryonic stem cell lines reveals culture-induced copy number changes and loss of heterozygosity. Nat Biotechnol. 2010; 28(4): 371–377. doi: 10.1038/nbt.1615 20351689 10.1038/nbt.1615

[39] Nikitina VA, Osipova EY, Katosova LD, Rumyantsev SA, Skorobogatova EV, Shamanskaya TV, et al Study of Genetic Stability of Human Bone Marrow Multipotent Mesenchymal Stromal Cells. . 2011; 150(5): 627–631. 22235401 10.1007/s10517-011-1207-1

