# Correction: Autologous and not allogeneic adipose-derived stem cells improve acute burn wound healing

**DOI:** 10.1371/journal.pone.0238935

**Published:** 2020-09-03

**Authors:** Yu-Wei Chang, Yi-Chia Wu, Shu-Hung Huang, Hui-Min David Wang, Yur-Ren Kuo, Su-Shin Lee

After publication of this article [1], concerns were raised regarding the lack of a representative image from the wound closure assay, and the precision and resolution of the figures. The minimal dataset was also not included for this published article as indicated in the Data Availability statement.

The complete underlying data for the Figs 1, 3, and 4 and the data underlying the Supporting Information tables has been provided within the Supporting Information file (S1 File).

In addition, in the discussion of the article cited as reference 15 in the text[2], the authors incorrectly stated that “…autologous ADSCs enhanced wound healing in patients with cutaneous radiation syndrome”. The authors would like to clarify that the study reported was performed in a minipig model, and not in patients.

PLOS ONE re-assessed this article in light of issues about the reported results raised post-publication and confirmed that the article meets PLOS ONE’s publication criteria, that the article as reported is valid and no further actions are required.

The authors apologize for the errors within the published article.

## Supporting information

S1 File Underlying data. (ZIP)
