# Supplementary material for: Autologous and not allogeneic adipose-derived stem cells improve acute burn wound healing
Source: PLoS One. 2018 May 22;13(5):e0197744. doi: 10.1371/journal.pone.0197744 (PMC5963767; doi:10.1371/journal.pone.0197744)
Supplement: S1 Table — (DOCX) [file pone.0197744.s001.docx]

**Supporting information**

| **S1 Table. Wound healing rate of Autologous ADSC VS Allogenic ADSC** | | | | | | | |
| --- | --- | --- | --- | --- | --- | --- | --- |
| **Healing Rate%** | Day 2 | Day 5 | Day 7 | Day 9 | Day 11 | Day 13 | Day 15 |
| **Control** | 14.25±1.03 | 29.60±1.83 | 43.52±1.05 | 60.02±1.38 | 73.00±1.16 | 82.67±1.17 | 91.41±0.41 |
| **Auto Center*#** | 16.40±1.93 | 37.78±1.49 | 54.80±0.78 | 78.10±1.44 | 87.33±0.76 | 94.90±0.59 | 98.92±1.00 |
| **Allo Center** | 14.58±1.13 | 31.24±1.62 | 44.16±1.22 | 58.50±0.73 | 70.80±1.20 | 79.51±1.43 | 89.92±0.79 |

Mean ±S.E.M; *p* values were calculated by *paired t- test.*

Number of animals = 3; Number of replicates = 6.
*Significant different from Control (*p*<0.05)

#Significant different from Allo Center (*p*<0.05)
