# Supplementary material for: Autologous and not allogeneic adipose-derived stem cells improve acute burn wound healing
Source: PLoS One. 2018 May 22;13(5):e0197744. doi: 10.1371/journal.pone.0197744 (PMC5963767; doi:10.1371/journal.pone.0197744)
Supplement: S2 Table — (DOCX) [file pone.0197744.s002.docx]

**Supporting information**

| S2 Table. Wound healing rate of Injection at Center VS Injection at 0.5cm from wound edge | | | | | | | | |
| --- | --- | --- | --- | --- | --- | --- | --- | --- |
| Healing Rate% | Day 2 | Day 5 | | Day 7 | Day 9 | Day 11 | Day 13 | Day 15 |
| Control | 14.25±1.03 | | 29.60±1.83 | 43.52±1.05 | 60.02±1.38 | 73.00±1.16 | 82.67±1.17 | 91.41±0.41 |
| Auto Center | 16.40±1.93 | 37.78±1.49 | | 54.80±0.78 | 78.10±1.44 | 87.33±0.76 | 94.90±0.59 | 98.92±1.00 |
| Auto 0.5cm*^#&^ | 16.41±1.87 | 38.98±1.34 | | 57.86±1.55 | 80.72±0.63 | 93.04±0.30 | 98.150.06 | 100±0.06 |
| Allo Center | 14.58±1.13 | 31.24±1.62 | | 44.16±1.22 | 58.50±0.73 | 70.80±1.20 | 79.51±1.43 | 89.92±0.79 |
| Allo 0.5cm^§^ | 16.71±0.58 | 32.19±1.65 | | 46.24±1.90 | 58.17±1.06 | 71.71±0.65 | 82.85±0.69 | 90.90±0.45 |

Mean ±S.E.M; p values were calculated by paired t- test.

Number of animals = 3; Number of replicates = 6.

*Significant different from Control (p<0.05)

#Significant different from Auto Center (p<0.05)

&Significant different from Allo 0.5cm (p<0.05)

§Significant different from Allo Center (p<0.05)
