# Supplementary material for: Correction: Autologous and not allogeneic adipose-derived stem cells improve acute burn wound healing
Source: PLoS One. 2020 Sep 3;15(9):e0238935. doi: 10.1371/journal.pone.0238935 (PMC7470251; doi:10.1371/journal.pone.0238935)
Supplement: S1 File — (ZIP) [file pone.0238935.s001.zip › Picture of the rat back wound.pptx]

## Slide 1
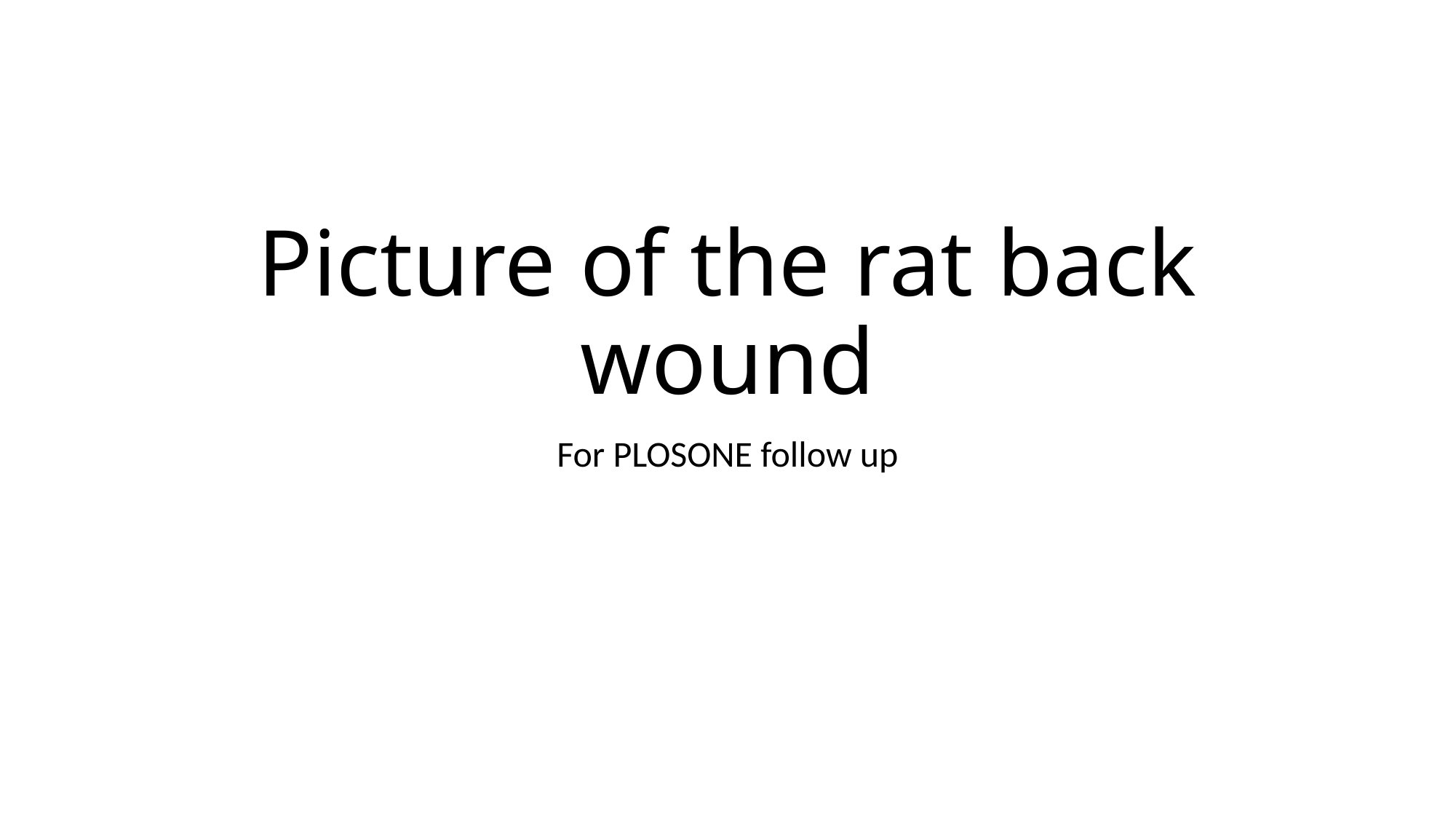

# Picture of the rat back wound
For PLOSONE follow up

## Slide 2
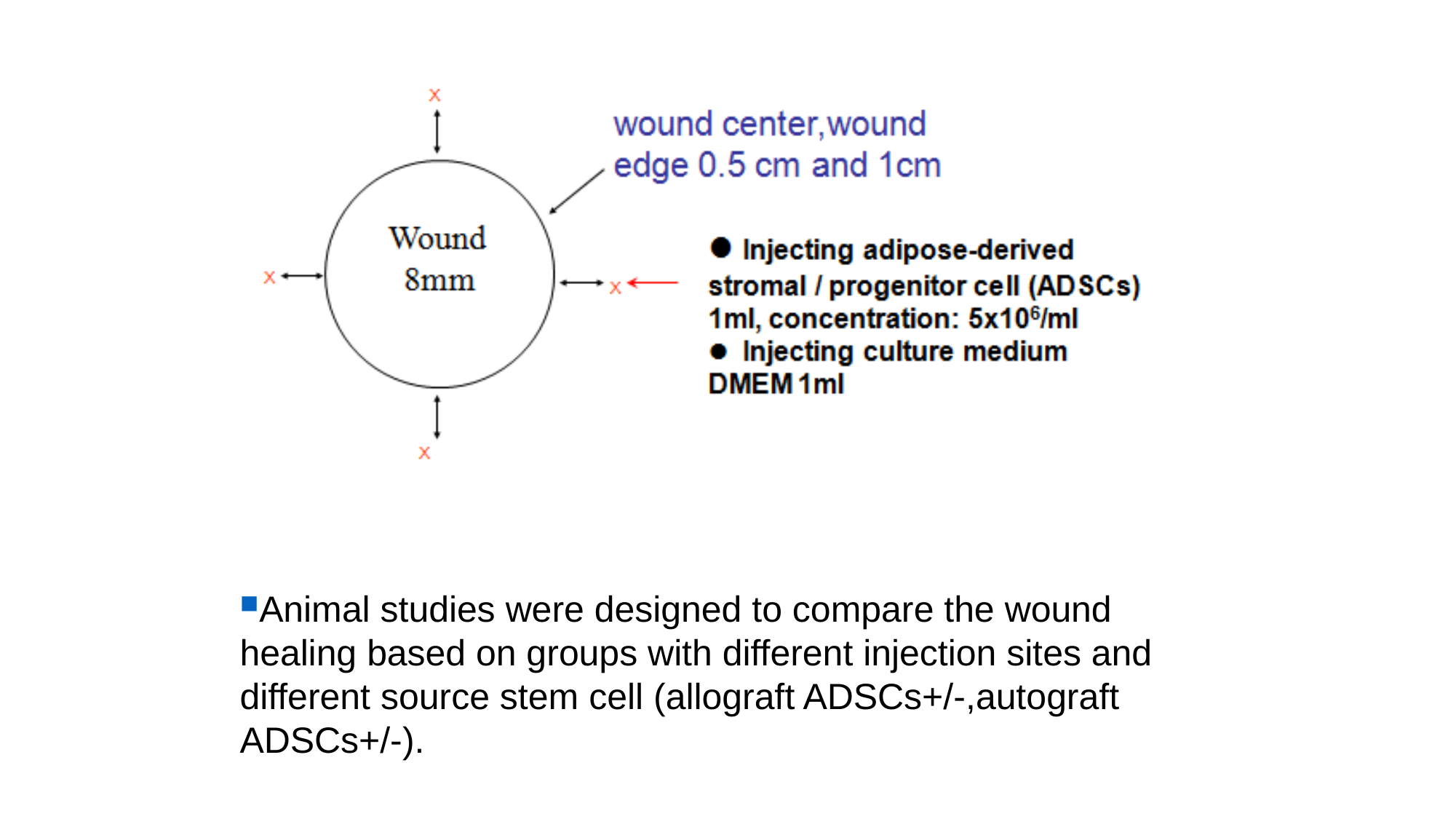

Animal studies were designed to compare the wound healing based on groups with different injection sites and different source stem cell (allograft ADSCs+/-,autograft ADSCs+/-).

## Slide 3
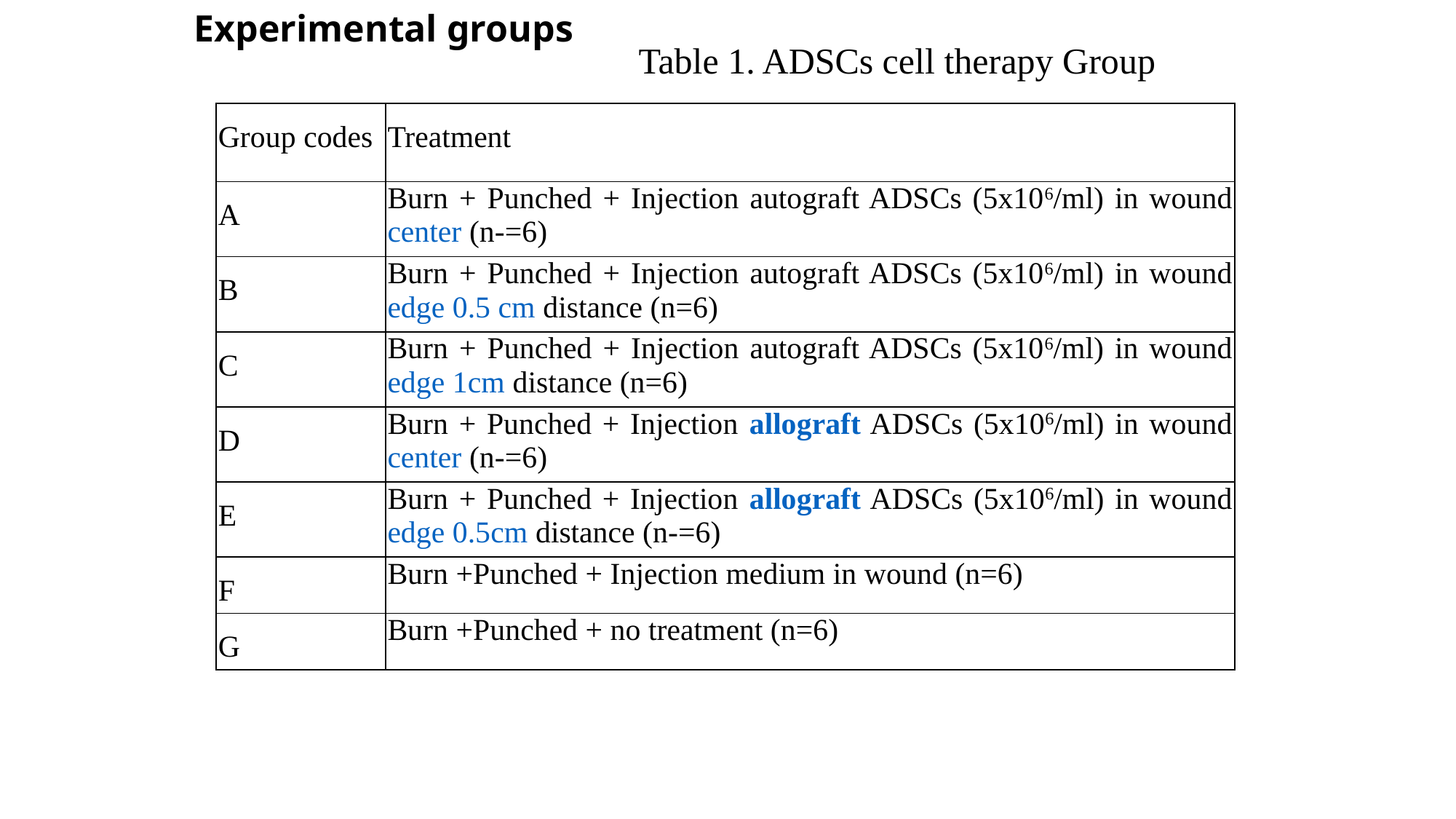

Experimental groups
Table 1. ADSCs cell therapy Group
| Group codes | Treatment |
| --- | --- |
| A | Burn + Punched + Injection autograft ADSCs (5x10^6^/ml) in wound center (n-=6) |
| B | Burn + Punched + Injection autograft ADSCs (5x10^6^/ml) in wound edge 0.5 cm distance (n=6) |
| C | Burn + Punched + Injection autograft ADSCs (5x10^6^/ml) in wound edge 1cm distance (n=6) |
| D | Burn + Punched + Injection allograft ADSCs (5x10^6^/ml) in wound center (n-=6) |
| E | Burn + Punched + Injection allograft ADSCs (5x10^6^/ml) in wound edge 0.5cm distance (n-=6) |
| F | Burn +Punched + Injection medium in wound (n=6) |
| G | Burn +Punched + no treatment (n=6) |

## Slide 4
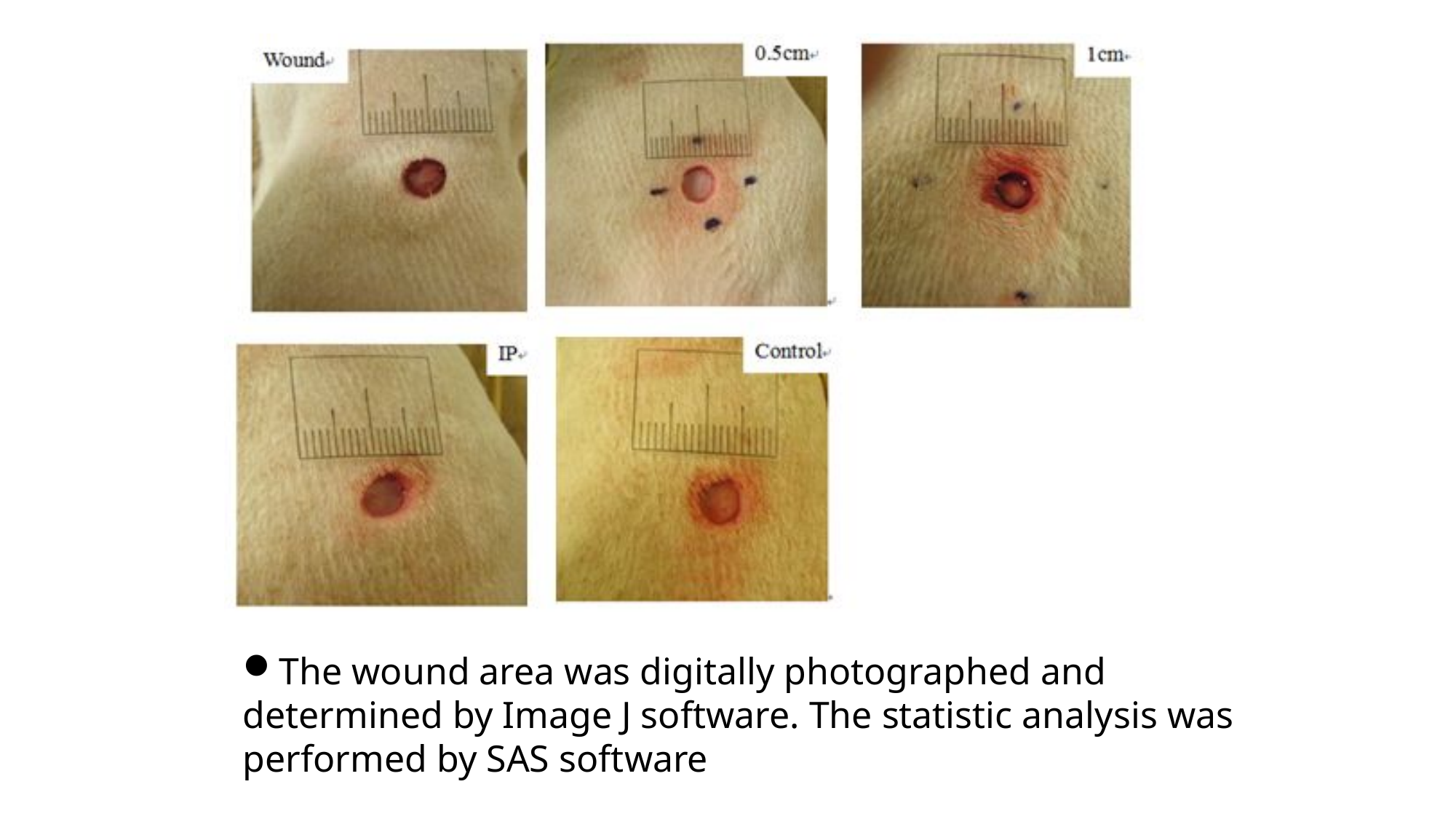

The wound area was digitally photographed and determined by Image J software. The statistic analysis was performed by SAS software

## Slide 5
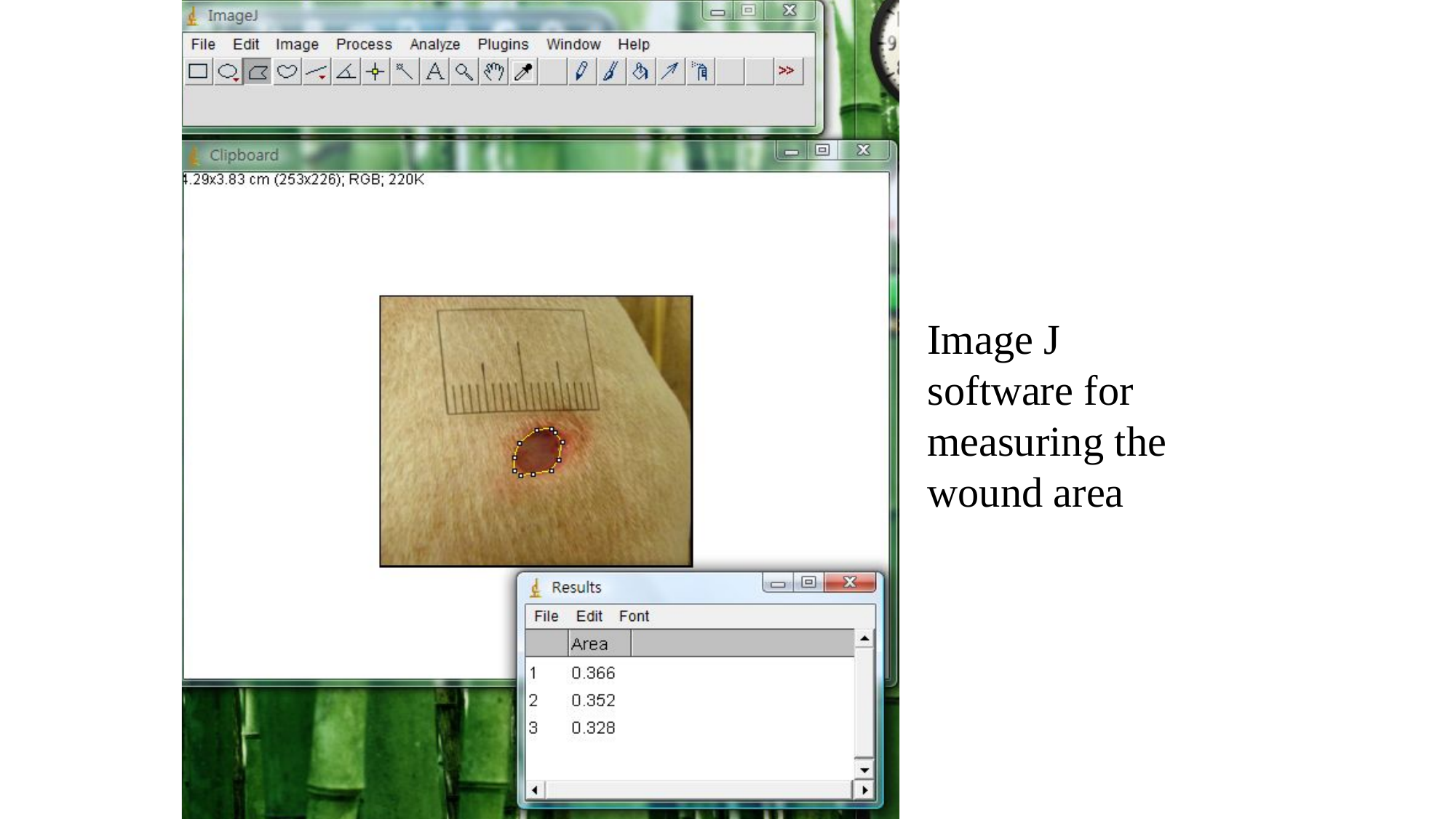

Image J software for measuring the wound area

## Slide 6
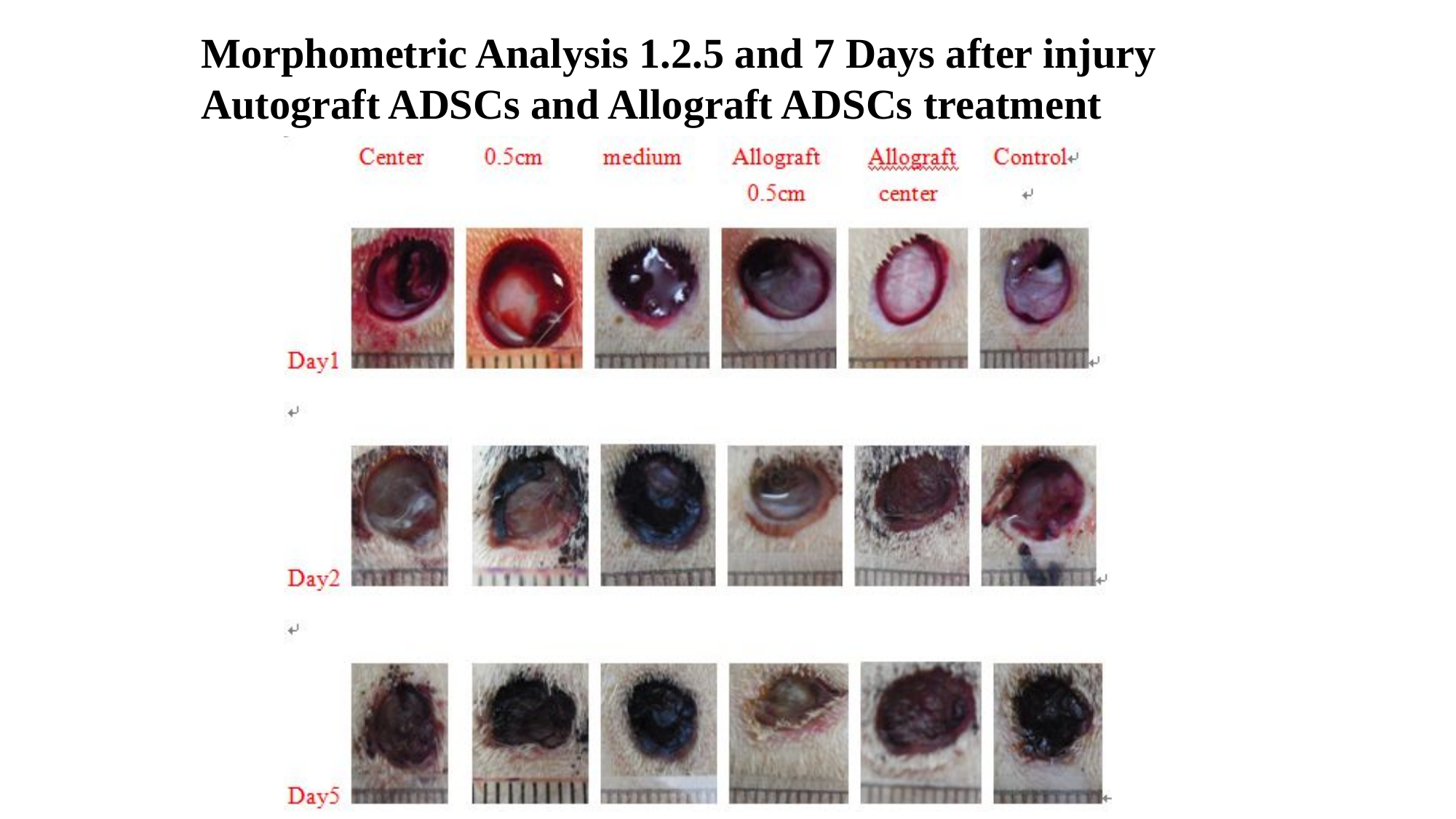

Morphometric Analysis 1.2.5 and 7 Days after injury
Autograft ADSCs and Allograft ADSCs treatment

## Slide 7
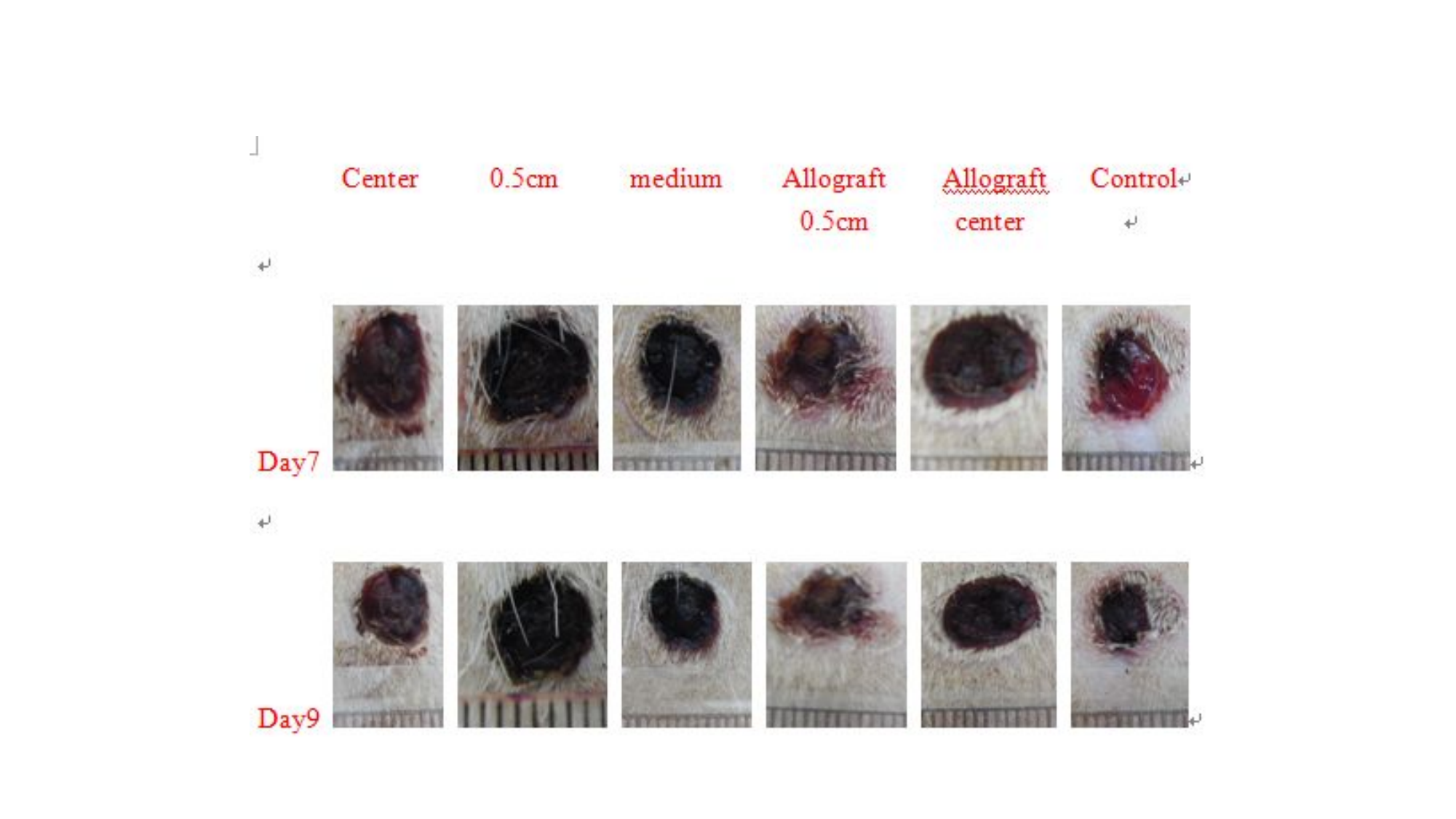

## Slide 8
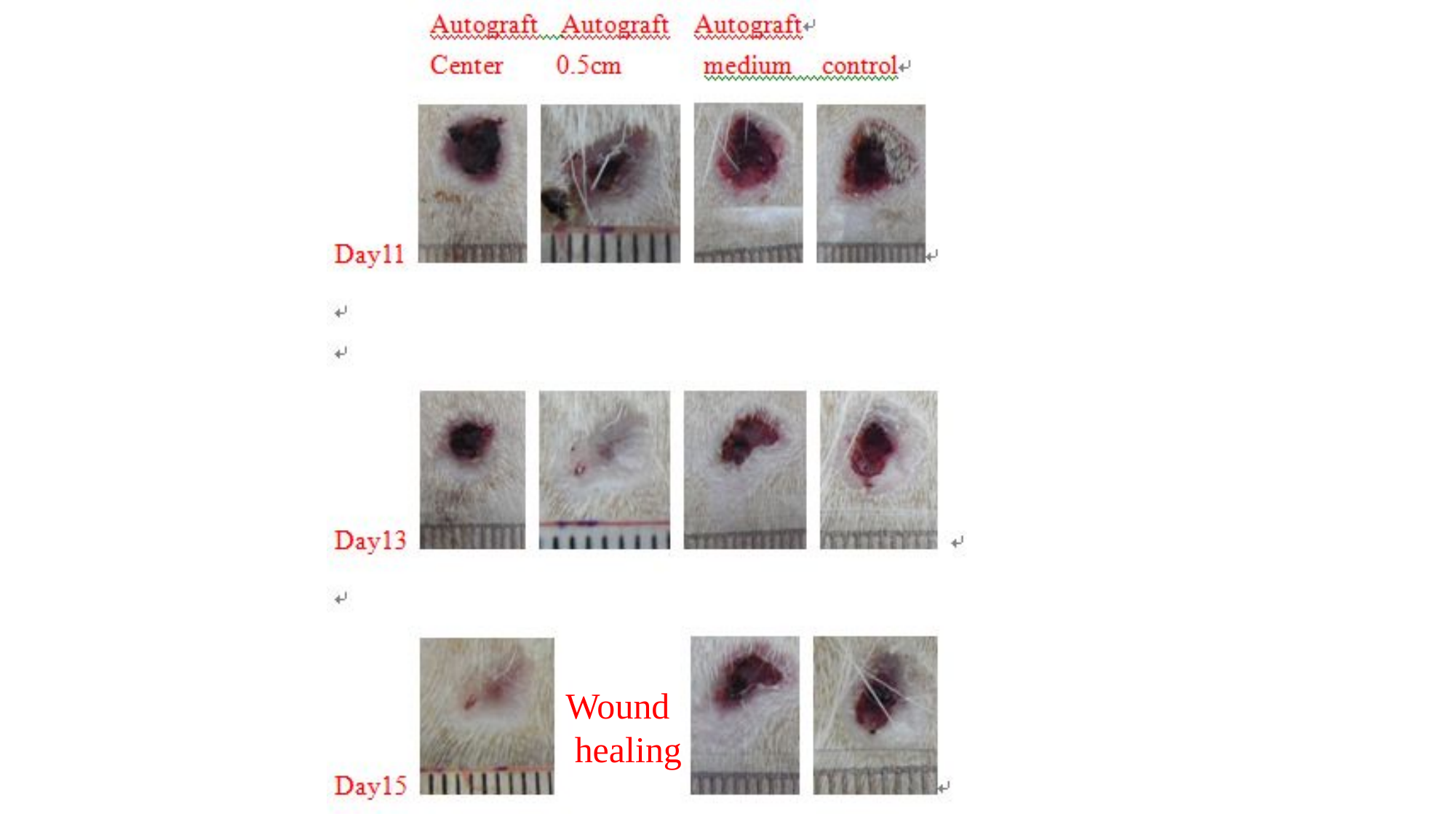

Wound
 healing

## Slide 9
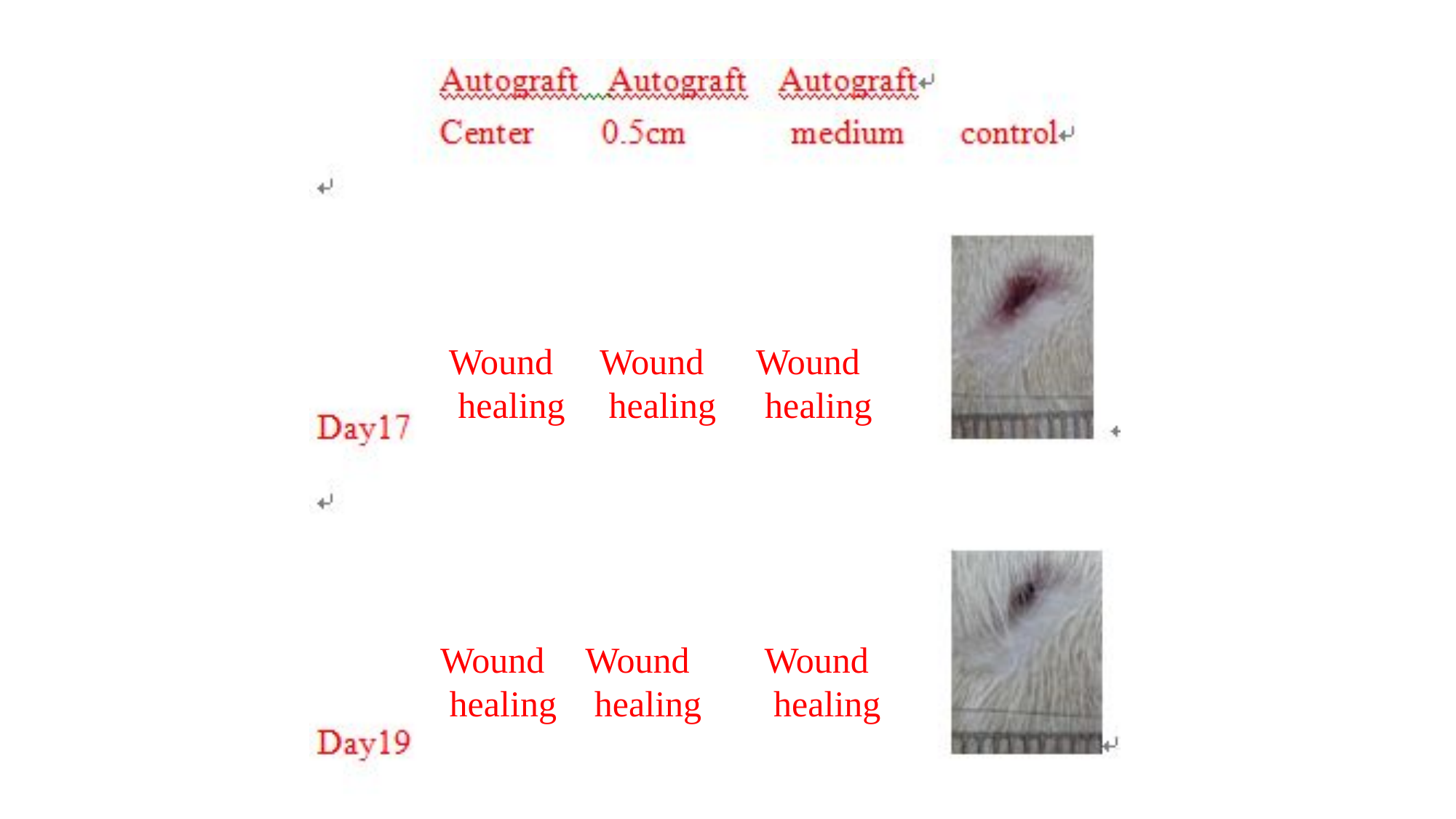

Wound
 healing
Wound
 healing
Wound
 healing
Wound
 healing
Wound
 healing
Wound
 healing
